# A novel adult plant leaf rust resistance gene *Lr2K38* mapped on wheat chromosome 1AL

**DOI:** 10.1002/tpg2.20061

**Published:** 2020-11-10

**Authors:** Suraj Sapkota, Mohamed Mergoum, Ajay Kumar, Jason D. Fiedler, Jerry Johnson, Dan Bland, Benjamin Lopez, Steve Sutton, Bikash Ghimire, James Buck, Zhenbang Chen, Stephen Harrison

**Affiliations:** ^1^ Institute of Plant Breeding, Genetics, and Genomics University of Georgia Griffin Campus Griffin GA 30223 USA; ^2^ Department of Crop and Soil Sciences University of Georgia Griffin Campus Griffin GA 30223 USA; ^3^ Department of Plant Sciences North Dakota State University Fargo ND 58102 USA; ^4^ USDA‐ARS, Cereal Crops Research Unit, Edward T. Schafer Agricultural Research Center Fargo ND 58102 USA; ^5^ Department of Plant Pathology University of Georgia Griffin Campus Griffin GA 30223 USA; ^6^ School of Plant, Environmental and Soil Sciences Louisiana State University Baton Rouge LA 70803 USA

## Abstract

Soft red winter wheat (SRWW) cultivar AGS 2038 has a high level of seedling and adult plant leaf rust (LR) resistance. To map and characterize LR resistance in AGS 2038, a recombinant inbred line (RIL) population consisting of 225 lines was developed from a cross between AGS 2038 and moderately resistant line UGA 111729. The parents and RIL population were phenotyped for LR response in three field environments at Plains and Griffin, GA, in the 2017–2018 and 2018–2019 growing seasons, one greenhouse environment at the adult‐plant stage, and at seedling stage. The RIL population was genotyped with the Illumina iSelect 90K SNP marker array, and a total of 7667 polymorphic markers representing 1513 unique loci were used to construct a linkage map. Quantitative trait loci (QTL) analysis detected six QTL*, QLr.ags‐1AL, QLr.ags‐2AS, QLr.ags‐2BS1, QLr.ags‐2BS2*, *QLr.ags‐2BS3*, and *QLr.ags‐2DS*, for seedling and adult plant LR resistance. Of these, the major adult plant leaf rust resistance QTL, *QLr.ags‐1AL*, was detected on all field and greenhouse adult plant tests and explained up to 34.45% of the phenotypic variation. *QLr.ags‐1AL*, tightly flanked by *IWB20487* and *IWA4022* markers, was contributed by AGS 2038. Molecular marker analysis using a diagnostic marker linked to *Lr59* showed that *QLr.ags‐1AL* was different from *Lr59*, the only known LR resistance gene on 1AL. Therefore, the QTL was temporarily designated as *Lr2K38*. *Lr2K38‐*linked marker *IWB20487* was highly polymorphic among 30 SRWW lines and should be useful for selecting the *Lr2K38* in wheat breeding programs.

**Abbreviations**
APR: Adult plant resistance
ASR: All‐stage resistance
BLUP: Best linear unbiased prediction
IT: Infection type
LOD: logarithm of odds
LR: Leaf rust
*Lr* genes: leaf rust resistance genes
MAS: Marker‐assisted selection
PCR: Polymerase chain reaction
QTL: Quantitative trait locus
RIL: Recombinant inbred line
SNP: Single Nucleotide Polymorphism
SRWW: Soft red winter wheat
UGA‐SGBP: University of Georgia Small Grain Breeding Program.

## 1 INTRODUCTION

Leaf rust (LR), caused by the biotrophic fungal pathogen *Puccinia triticina* Erikss is one of the major foliar diseases of wheat (*Triticum aestivum* L.) worldwide causing significant yield reduction. Yield losses of up to 40% have been reported due to LR; however, losses can vary depending upon the cultivar, LR severity, time of initial infection, rate of disease development, and disease duration (Kolmer, Jin, & Long, 2007; Zhao et al., 2008). Although several management practices, including the use of fungicides, are available and found effective to control wheat LR, the use of genetic resistance is undoubtedly the best and most preferred method to control LR disease (Ghimire et al., 2020; Kolmer, 1996). To date, 79 leaf rust resistance genes (*Lr* genes) have been cataloged (Qureshi et al., 2018) and 249 QTL conferring LR resistance have been reported (Pinto da Silva et al., 2018). However, the majority of the genes and/or QTL reported confer race‐specific resistance and can be easily overcome by changes in the pathogen population. Therefore, a continuous search for novel sources of LR resistance is key to combating this disease.

Resistance to rusts in wheat is broadly classified into two types: 1) race‐specific resistance which is normally detected at the seedling stage, and 2) race non‐specific resistance which is detected at the adult plant stage (Johnson, 1988; Kolmer, 2013). Seedling resistance, also known as all‐stage resistance (ASR), is detected at an early growth stage (seedling) and effective throughout the host life cycle (Line & Chen, 1995). Seedling resistance, often effective against certain *P. triticina* races carrying corresponding avirulence genes, frequently confers a high level of resistance by development of a hypersensitive reaction in host plants (Kolmer, 2005). Of the 79 reported *Lr*‐genes, 64 are race‐specific and confer LR resistance at the seedling stage (Pinto da Silva et al., 2018). A major limitation of race‐specific resistance genes is that they are short‐lived and mutation and/or recombination in *P. triticina* populations can easily overcome the seedling resistance (Ghimire et al., 2020; Kolmer, 2005, 2013). For instance, two seedling *Lr*‐genes, *Lr10* and *Lr16*, that were deployed in Canadian wheat cultivar ‘Selkirk’ were ineffective after the evolution of new virulent *P. triticina* races (McCallum et al., 2016). Pyramiding multiple seedling *Lr*‐genes could possibly prolong the duration of resistance cultivars; however, the occurrence of 40–60 *P. triticina* races each year in the USA poses a great challenge to this approach (Kolmer, 2005).

Adult plant resistance (APR), also known as partial resistance or slow‐rusting resistance, has been reported to be more durable than seedling resistance (Bolton, Kolmer, & Garvin, 2008; Kolmer, 1996, 2005). Currently, there are 15 formally cataloged *Lr*‐genes that confer APR. Of these, seven (*Lr12*, *Lr13*, *Lr22a/b*, *Lr35*, *Lr37*, *Lr48*, and *Lr49*) and eight (*Lr34*, *Lr46*, *Lr67*, *Lr68*, *Lr74*, *Lr75*, *Lr77*, and *Lr78*) are reported to be race‐specific and race non‐specific APR genes, respectively (Pinto da Silva et al., 2018; Zhang et al., 2019). Three of eight race non‐specific APR genes, *Lr34*, *Lr46*, and *Lr67*, are unique and more valuable for plant breeding because they are pleiotropic and confer partial resistance to all three rusts (leaf, stripe, and stem rusts) and powdery mildew caused by *Blumeria graminis* f. sp. *tritici* (Herrera‐Foessel et al., 2014; Pinto da Silva et al., 2018; William, Singh, Huerta‐Espino, Islas, & Hoisington, 2003).

Linkage mapping using bi‐parental populations and association mapping using diversity panels have been used in identifying QTL for LR resistance (Qureshi et al., 2018; Sapkota et al., 2019a, 2019b). However, the majority of LR resistance QTL have been identified using bi‐parental crosses (Pinto da Silva et al., 2018). Advancement in Illumina iSelect genotyping platform, 9K, 90K, and 820K SNP arrays, and the recently released Chinese Spring wheat reference genome, RefSeq‐v1.0 (https://www.wheatgenome.org/), have enabled more precise location of LR resistance QTL in the wheat genome and comparison of QTL with previously reported genes/QTL using wheat consensus and physical maps (Cavanagh et al., 2013; International Wheat Genome Sequencing Consortium 2018; Wang et al., 2014; Winfeld et al., 2016).

**Core Ideas**

- Soft red winter wheat cultivar AGS 2038 has a high level of seedling and adult plant leaf rust (LR) resistance
- In this study, we mapped and characterized a novel and important adult plant LR resistance gene, *Lr2K38*, on 1AL wheat chromosome
- *Lr2K38* linked markers were converted into allele‐specific primers and tested on wheat lines to determine their efficacy for marker‐assisted selection
- *Lr2K38*‐linked marker, *IWB20487*, should be useful for deploying the *Lr2K38* gene in wheat breeding programs

The University of Georgia Small Grain Breeding Program (UGA‐SGBP) has long been dedicated to the development of wheat cultivars which are suitable to the Southeastern region of the US and, to‐date, more than 30 wheat cultivars have been released. AGS 2038 is one of the soft red winter wheat (SRWW) cultivars released in 2011 by the UGA‐SGBP and licensed to AGSouth Genetics Company for its high yield, good test weight, medium maturity, and resistance to current biotypes of Hessian fly in Georgia, USA. Additionally, we observed that AGS 2038 exhibited high levels of seedling and adult plant LR resistance in greenhouse and field tests since its release. The objectives of this study were to map genomic loci conferring seedling and adult plant LR resistance in AGS 2038 using a high‐density single nucleotide polymorphism (SNP)‐linkage map and to develop user‐friendly markers that can be used in marker‐assisted selection (MAS) to develop LR resistant wheat cultivars.

## 2 MATERIALS AND METHODS

### 2.1 Mapping population development

A mapping population consisting of 225 RILs (F_6_) was developed from a cross between LR resistant and moderately resistant SRWW lines, AGS 2038 and UGA 111729, respectively. AGS 2038, developed by the UGA‐SGBP and released by the Georgia Agricultural Experiment Station in 2011, was derived from a cross between GA 961581 and PIO 26R61, both of which possess good LR resistance at adult plant stage. UGA 111729 is an elite wheat breeding line also developed by UGA‐SGBP. Additionally, a set of 30 SRWW cultivars and elite breeding lines were selected and used to test the polymorphism of the resistance‐linked markers (Supplemental Table S1).

### 2.2 Phenotyping

The parents and the RIL population were evaluated for adult plant LR reaction in the field at Plains, GA during the 2017–2018 season (APR‐PL18) and at two locations, Plains and Griffin, GA during the 2018–2019 growing seasons (APR‐PL19 and APR‐GRF19). The Griffin Experiment Station (Bledsoe farm) is located close to the UGA, Griffin Campus, whereas the Plains Experiment Station is located 105 miles south from Griffin, GA. These locations represent quite different ecological environments in GA. The plant materials were arranged in a randomized complete block design (RCBD) with two replications per location. Each entry was planted as a single row plot, 1‐m long and 25 cm apart and approximately 3 gm of seeds were sown in each plot. The parents, AGS 2038 and UGA 111729, were planted after every 20 rows as checks. A highly susceptible SRWW cultivar, SS520, was planted around the research plot area as a rust spreader and to uniformly increase the LR epidemics throughout the trials. A few plants of susceptible spreader, SS520, were inoculated with *P. triticina* race MFGKG, the current prevalent *P. triticina* race in Georgia (Sapkota et al., 2019b), in the growth chamber and when the symptoms were visible, the infected plants were transplanted in the middle of the spreader plots to disperse the urediniospores in the field plots. The LR severities (the percentage of leaf tissue infected) on the parents and RILs were recorded using the modified Cobb scale (Peterson, Campbell, & Hannah, 1948) when the moderately resistant parent (UGA 111729) and most susceptible RILs had the highest level of LR severity.

The parents and RILs were also evaluated under greenhouse conditions for adult plant reaction in 2018 (APR‐GH18) at UGA, Griffin Campus, Griffin, GA. Two seeds of each parent and RIL were planted in cone‐tainers (Stuewe and Sons, Inc) filled with SunGro growing mix soil (Sun Gro Horticulture Distribution Inc.) and placed in RL98 racks. Three cone‐tainers were planted for each line with six plants in total. AGS 2038 and UGA 111729 were planted in each rack as checks. After germination, the plant materials were placed in a cold room set at 5°C for six weeks for vernalization, and subsequently transferred to the greenhouse bench set at 20 °C with a 15 h of photoperiod. The plant materials were inoculated at the anthesis stage when flag leaves were fully emerged (Feekes 10.5) with the *P. triticina* race MFGKG. Urediniospores for adult plant testes in the greenhouse were collected from susceptible wheat cultivar SS520 in the field (Plains, GA) and maintained in the growth chamber. Inoculation was done as described in Sapkota et al. (2019b). The RILs and parental lines were evaluated for LR reaction two weeks post inoculation using the standard 0–4 infection type (IT) scale (Long & Kolmer, 1989) and LR severity. However, only the severity data was used for subsequent analysis due to greater variation among the RILs.

For seedling or all‐stage resistance (ASR‐GH20) test, plant material preparation, inoculation, disease scoring, and statistical analysis of the phenotypic data was done as described in Sapkota et al. (2019a, 2019b). *P. triticina* race, MFGKG, which is a current common race in GA (Sapkota et al., 2019b) was used. The seedling test was conducted in the growth chamber at the University of Georgia, Griffin Campus, using RCBD with 3 replications.

### 2.3 Statistical analysis of the adult plant response data

Analysis of variance (ANOVA) was performed using the general linear model procedure in SAS version 9.4 (SAS Institute Inc., 2017) to estimate the effects of different variables in the experiments. Pearson correlation coefficients (*r*) for the LR severity scores were calculated to determine the consistency of LR response across environments using the pairs.panel function in R package “psych” (Revelle, 2017). The best linear unbiased predictions (BLUPs) values were calculated for LR severity across all environments using the “lme4” package in R (Bates, Machler, Bolker, & Walker, 2015; R Core team, 2016). Genotype, environment, genotype by environment, and replication were all treated as random effects in the model. For individual environment, only genotype and replication were used in the model and both were treated as random variables to calculate BLUPs. BLUP values calculated for each genotype across and within environments were used in the subsequent QTL analysis as phenotypic data (Supplemental Table S6). Broad sense heritability (*H^2^*) was calculated across all environments and for each single environment to determine the effect of genotype on phenotype. *H^2^* was calculated according to the equations $H^{2}=\sigma_{G}^{2}\;/\left(\sigma_{G}^{2}+\sigma_{e}^{2}/r\right)$ and $H^{2}=\sigma_{G}^{2}\;/\left(\sigma_{G}^{2}+\sigma_{GE}^{2}/r+\sigma_{e}^{2}/er\right)$ for individual environment and across all environments, respectively, where $\sigma_{G}^{2}$ is the genotypic variance, $\sigma_{GE}^{2}$ is the genotype by environment interaction variance, $\sigma_{e}^{2}$ is the residual variance, *e* is the number of environment, and *r* is the number of replications in each environment.

### 2.4 SNP genotyping

High quality genomic DNA of the two parents and RILs were sent to the United States Department of Agriculture‐Agricultural Research Service, Small Grain Genotyping laboratory at Fargo, ND, for 90K iSelect SNP genotyping (Wang et al., 2014). A total of 81,587 SNP markers were produced by the Illumina iSelect 90K SNP assay and alleles were called using GenomeStudio 2.0 software. Necessary corrections were made manually to minimize errors related to allele calls. A total of 8800 markers were polymorphic between the parents, but 1,133 markers were removed for various reasons including: 1) inconsistent results from three replications of each parental line; 2) overlapping clustering; 3) > 20% missing data, and 4) highly distorted markers i.e., that did not fit a 1:1 segregation ratio. Finally, 7667 SNP markers representing 1513 unique loci were used for linkage map construction and QTL analysis (Supplemental Table S7).

### 2.5 Linkage map construction and QTL analysis

Linkage maps were created by first grouping markers with the Minimum Spanning Tree algorithm described by Wu, Bhat, Close, and Lonardi (2008) and implemented in the R package “ASMap”. Groups with more than 20 markers were converted into “.loc” files for import into Joinmap 5 (van Ooijen, 2006). Individual population nodes were grouped again in Joinmap to generate a single grouping and a grouping consisting of sub‐groups of approximately 200 markers based on LOD. Single groups were ordered based on the Maximum Likelihood algorithm using default settings. Sub‐groups were ordered using the Regression algorithm utilizing a starting order from the single‐group Maximum Likelihood results and Kosambi distance calculation. Sub‐group and single‐group maps were combined together with the published consensus map (Wen et al., 2017) via a linear programming algorithm with the R package “LPmerge”. All maps were given equal weighting and constructed with max.interval parameter varying from 1 to 6. The resulting maps with the lowest mean root mean squared error were used for QTL mapping.

QTL analysis was performed using the “BIP” (QTL mapping in bi‐parental populations) function in QTL IciMapping (Meng, Li, Zhang, & Wang, 2015). The inclusive composite interval mapping of additive (ICIM‐ADD) QTL method with a walk speed of 1.0 cM and 0.001 probability in stepwise regression was chosen for QTL detection. A logarithm of odds (LOD) value of 3.0 was chosen to declare significant QTL, and the LOD value was calculated from 1000 permutations with type I error of 0.01. The graphical representation of the genetic linkage map was generated using MapChart (Voorrips, 2002).

To determine the physical position of the QTL detected in this study, the sequences of the SNP markers associated with the QTL were obtained from Triticeae Toolbox website (triticeaetoolbox.org) and used to BLAST the Chinese Spring (CS) reference genome sequence (IWGSC RefSeq v1.0). Once the physical position of the QTL region was confirmed, the candidate genes within the major QTL region were downloaded from the website https://urgi.versailles.inra.fr/download/iwgsc/IWGSC_RefSeq_Annotations/v1.0/. If multiple transcripts existed for a single gene with the same function, only the first transcript was considered.

### 2.6 Conversion of SNP markers and genotyping assays

SNP markers, *IWB204878* and *IWA4022*, that flanked the major QTL were converted to user‐friendly allele‐specific primers and tested on a set of wheat materials to determine their utility for MAS. Primer Express version 3.0.1 (Applied Biosystems, Foster City, CA) was used to design primers and probes. The primer pairs for each SNP were synthesized by Eurofins Genomic LLC (Louisville, KY) and are presented in Supplemental Table S2. Two TaqMan probes with distinct 5′ reporter fluorophores, 3′ minor grove binders (MGB), and 3′ nonfluorescent quenchers (NFQ) were synthesized and obtained from Applied Biosystems (Foster City, CA). The fluorogenic probe sequences were 5′‐VIC TGTTATTCTTCATCATCGC MGBNFQ‐3′ and 5′‐6FAM TGTTATTCTTCATCATTGC MGBNFQ‐3′ for *IWB20487* marker and 5′‐VIC ATTACTTTCCGAAGCGAGA MGBNFQ‐3′ and 5′‐6FAM TACTTTCCGAAGCAAG MGBNFQ‐3′ for *IWA4022* markers. Polymerase chain reaction (PCR) assays were set up as described by Barkley, Chenault‐Chamberlin, Wang, and Pittman (2010) with minor modifications. Briefly, 15 μl PCR reactions containing 1 × TaqMan Genotyping Master Mix (Applied Biosystems), 0.16 μM forward primer, 0.16 μM reverse primer, 0.4 μM Vic probe, 0.4 μM 6Fam probe, 10 ng/μl of DNA, and autoclaved ddH_2_O were set up. The PCR thermal‐cycling consisted of 1 cycle of 60 °C for 30 s, 1 cycle of 95 °C for 10 min, 50 cycles of 95 °C for 15 s and 60 °C for 1 min, and a final cycle of 60 °C for 30 s. The end point fluorescence data were visualized with a QuantStudio 3 real‐time PCR system (Applied Biosystems).

### 2.7 Testing for presence of *Lr59* using the diagnostic marker

A Sequence Characterized Amplified Region marker, S15‐T3, diagnostic for *Lr59* (Marais, Kotze, & Eksteen, 2010) was used to determine its relationship with a major QTL detected in the present study. The S15‐T3 marker probe (forward primer = GTCACTTGCTTGAATTTAATG; reverse primer = TCCATAGCTGGTAGCTAGATG) amplified a 622 bp band diagnostic for *Lr59*. The genomic DNA from both parents, AGS 2038 and UGA 111729, was extracted using a modified CTAB method (Saghai‐Maroof, Soliman, Jorgensen, & Allard, 1984) and diluted to 50 ng/μl. The PCR reaction was carried out as described by Hao et al. (2008) and amplified products were separated in 1.5% agarose gel stained with ethidium bromide. A 100 bp DNA ladder (New England Biolabs, Inc.) was used to determine the size of the amplified products.

## 3 RESULTS

### 3.1 Phenotypic data analysis

**FIGURE 1:**
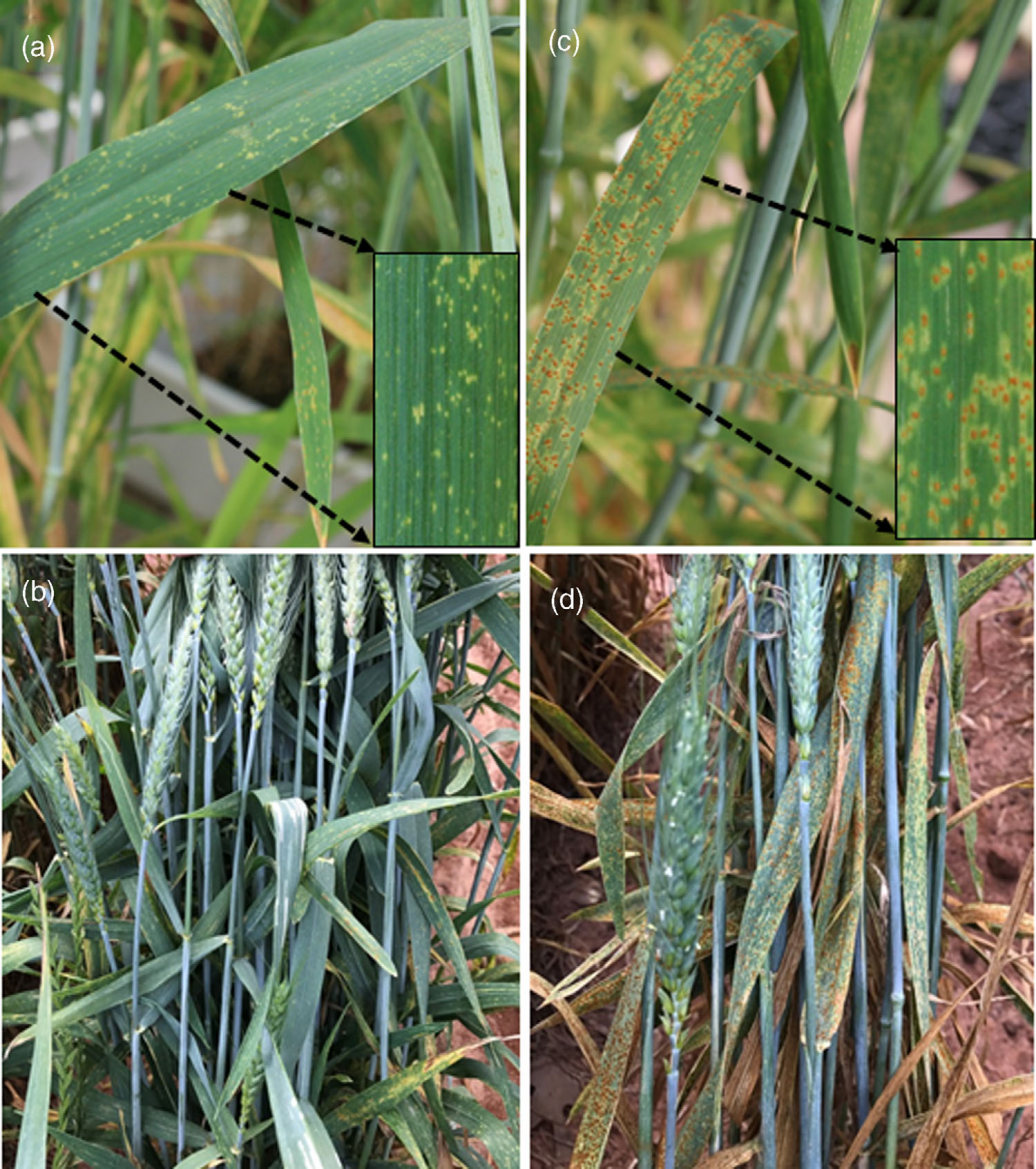
Adult plant leaf rust reaction of AGS 2038 (a and b) and UGA 111729 (c and d) under greenhouse (a and c) and field (b and d) environments

**FIGURE 2:**
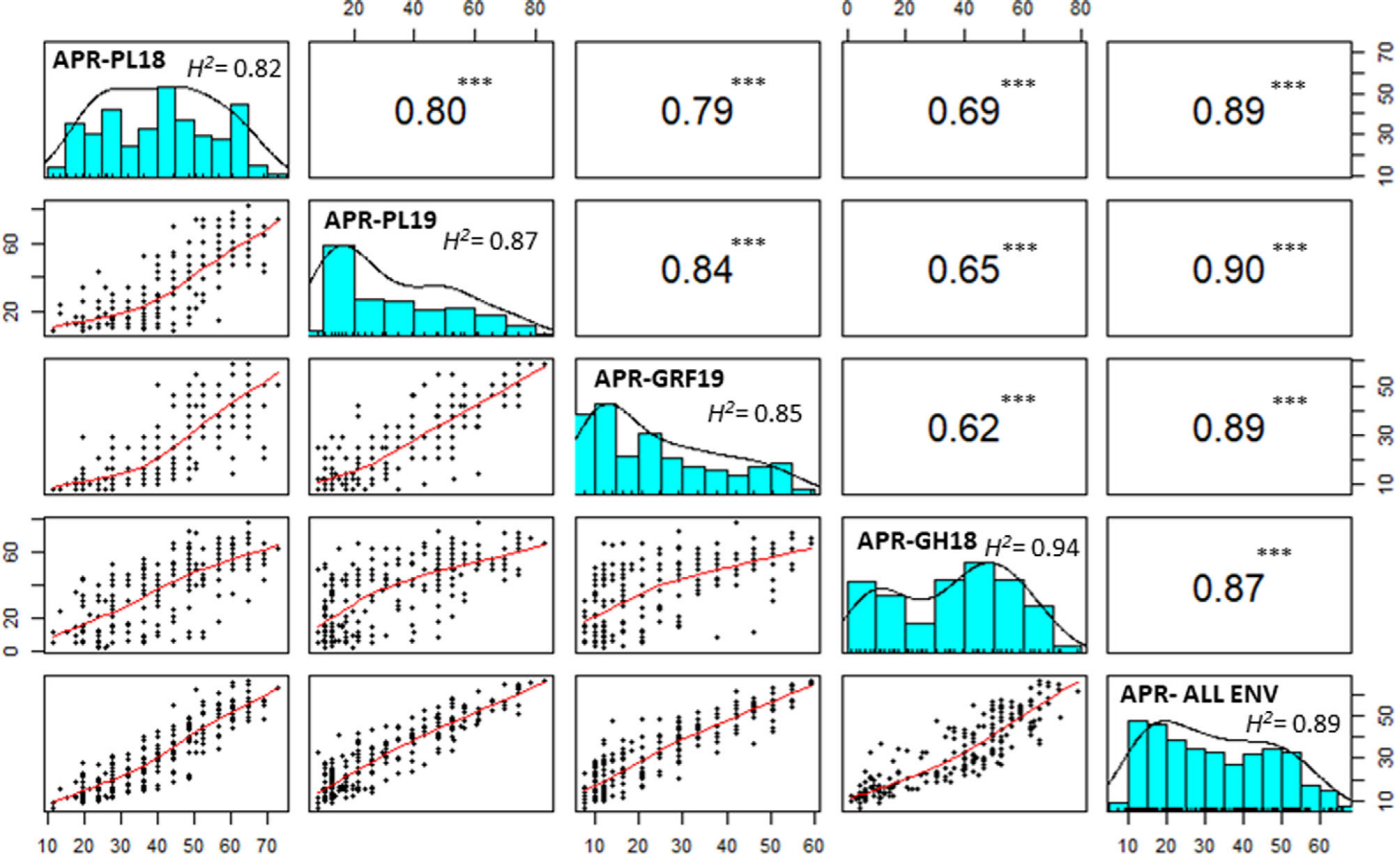
Trait distributions and correlation plots. The diagonal plots show the frequency distribution of leaf rust (LR) severity data of 225 recombinant inbred line (RIL) population in each environment (APR‐PL18 = field test at Plains in 2018; APR‐PL19 = field test at Plains in 2019; APR‐GRF19 = field test at Griffin in 2019; APR‐GH18 = greenhouse adult plant test in 2018; and APR‐ALL ENV = all environments combined adult plant test data). The panel above and below the diagonal represents Pearson's correlation coefficient and scatter plots, respectively. The broad‐sense heritability (*H^2^*) for each trait is shown in the diagonal histogram plots ***p < .0001

In both growing seasons, 2017–2018 and 2018–2019, AGS 2038 had LR severities of 5–10% demonstrating a highly resistant reaction while the other parent, UGA 111729, had LR severities of 40–50%. In the greenhouse adult plant test, AGS 2038 developed a hypersensitive reaction with small uredia (IT = ;1) and had LR severity of 10–20% whereas UGA 111729 developed medium‐sized uredia with chlorosis (IT = 3) and severity of 40–50%, demonstrating resistant and moderately resistant reactions, respectively (Figure 1). The RIL population segregated well for their reaction to LR in the field and greenhouse tests at the adult plant stage. The range of LR severity of RIL population in the field and greenhouse adult plant tests were 5 to 90%, 5 to 90%, 5 to 70%, and 0 to 80% in APR‐PL18, APR‐PL19, APR‐GRF19, and APR‐GH18 environments, respectively. Transgressive segregation indicated that both parents possessed different genes for LR resistance. The *H^2^* for LR severity was high for all four environments, with *H^2^* ranging from 0.82 to 0.94, indicating that non‐genetic effects were minimal on LR severity (Figure 2). The severity data among the four environments were highly correlated with values ranging from r = 0.62 (APR‐GRF19/APR‐GH18) to r = 0.84 (APR‐PL19/APR‐GRF19) and were highly significant (*p* < .0001) (Figure 2). ANOVA confirmed significant (*p* < .0001) variation among genotypes (RILs), environments, and genotype × environment interactions (Supplemental Table S3).

At the seedling test, AGS 2038 developed hypersensitive flecks with small sized uredia (IT = ;1) while UGA 111729 developed medium sized uredia with chlorosis (IT = 3) representing resistant and moderately resistant reactions, respectively. The RILs also segregated well and their reactions ranged from highly resistant to highly susceptible (Supplemental Table S4).

### 3.2 Construction of genetic linkage map

A genetic linkage map for the AGS 2038/UGA 111729 derived RIL population was generated using 7,667 polymorphic markers covering the whole wheat genome (Table 1). These markers represented 1,513 unique loci (Table 1). The genetic map consisted of 27 linkage groups representing all 21 wheat chromosomes. The B‐genome contained the greatest number of markers followed by the A and D‐genomes. The number of markers and unique loci in each linkage group ranged from 11 to 1208 and 10 to 206, respectively (Table 1). The map covered a total of 2881.46 cM distance with an average distance of 0.38 cM between adjacent markers. The A, B, and D‐genomes covered a total of 1164.27, 965.23, and 751.96 cM length with average map density (cM/marker) of 0.36, 0.26, and 1.05 cM, respectively (Table 1).

**TABLE 1:** Distribution of markers and marker density across linkage groups in AGS 2038/UGA 111729 derived recombinant inbred line population

Linkage group/Chromosome^a^	No. of Markers	No. of unique loci	Length (cM)	Average map density (cM/marker)	Average map density (cM/locus)
**Genome A**					
1A	623	106	67.27	0.11	0.63
2A	529	111	130.92	0.25	1.18
3A1	105	28	57.18	0.54	2.04
3A2	162	53	118.95	0.73	2.24
4A	357	99	167.25	0.47	1.69
5A	302	77	218.12	0.72	2.83
6A1	104	31	148.18	1.42	4.78
6A2	264	47	20.89	0.08	0.44
7A	781	127	235.51	0.30	1.85
**All**	**3227**	**679**	**1164.27**	**0.36**	**1.71**
**Genome B**					
1B	888	105	89.16	0.10	0.85
2B	1208	206	326.81	0.27	1.59
3B	206	53	107.21	0.52	2.02
4B	11	10	89.43	8.13	8.94
5B1	107	37	49.64	0.46	1.34
5B2	420	45	124.5	0.30	2.77
6B1	57	12	51.1	0.90	4.26
6B2	404	77	79.95	0.20	1.04
7B1	191	36	33.74	0.18	0.94
7B2	231	31	13.69	0.06	0.44
**All**	**3723**	**612**	**965.23**	**0.26**	**1.58**
**Genome D**					
1D	116	46	107.98	0.93	2.35
2D1	132	34	76.01	0.58	2.24
2D2	39	16	160.3	4.11	10.02
3D	150	29	145.83	0.97	5.03
4D	42	23	116.44	2.77	5.06
5D	102	27	18.17	0.18	0.67
6D	103	23	18.9	0.18	0.82
7D	33	24	108.33	3.28	4.51
**All**	**717**	**222**	**751.96**	**1.05**	**3.39**
**Whole Genome**	7667	1513	2881.46	0.38	1.90

^a^ The chromosomes are split into two linkage groups when the genetic distance or gap between the markers is > 50 cM.

### 3.3 QTL detection

**FIGURE 3:**
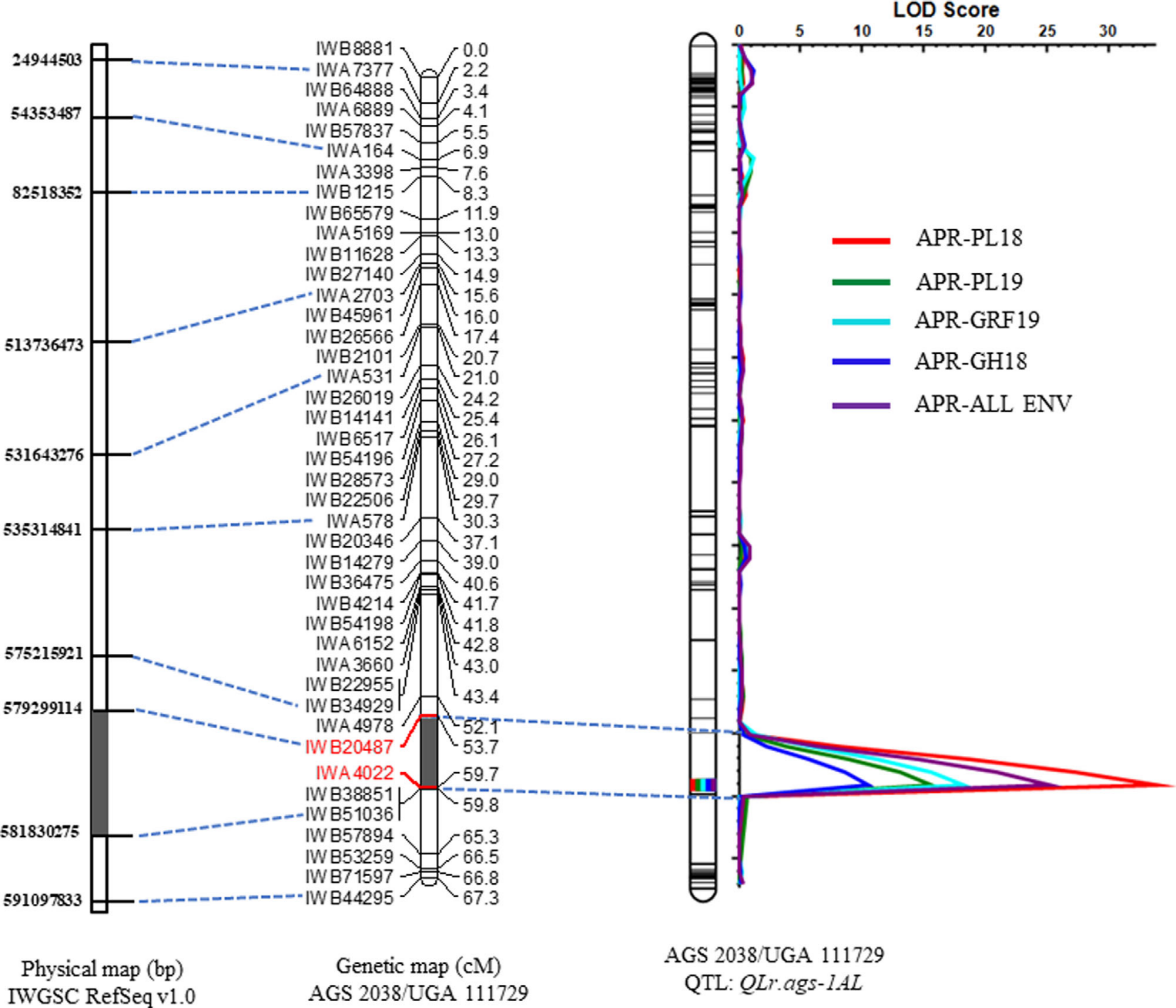
Genetic and physical map showing the location of major QTL, *QLr.ags‐1AL*, detected for adult plant leaf rust resistance in AGS 2038/UGA 111729 recombinant inbred line population. The QTL region is shown by grey solid region in the genetic and physical maps and QTL flanking markers are in red font

**FIGURE 4:**
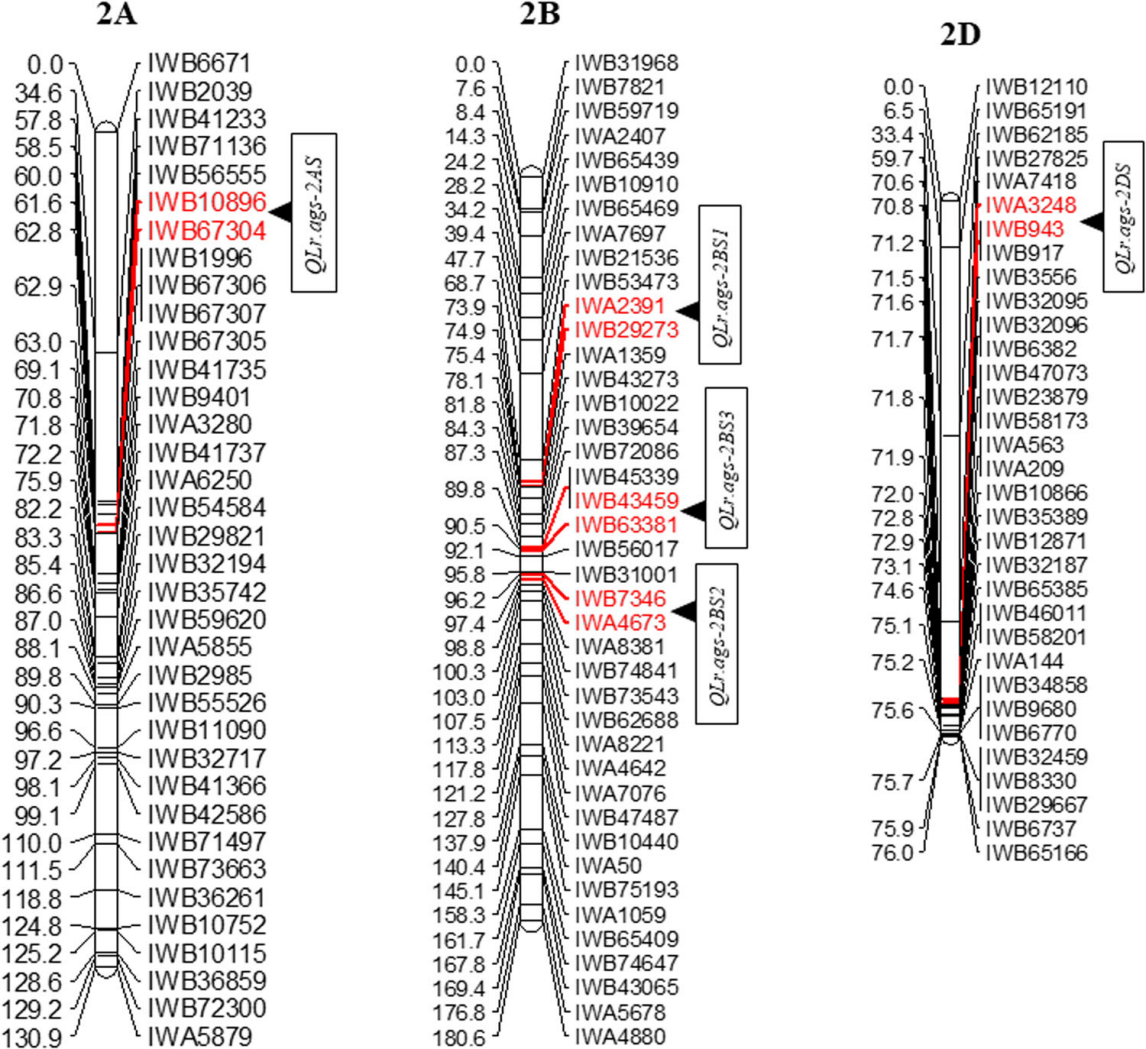
Linkage map showing seedling and adult plant resistance QTL detected on chromosomes 2A, 2B, and 2D in the AGS 2038/UGA 111729 recombinant inbred line population. The flanking markers for each QTL are in red font

The inclusive composite interval mapping (ICIM) in the AGS 2038/UGA 11729 RIL population detected six QTL for seedling and adult plant LR resistance on chromosomes 1A, 2A, 2B, and 2D (Table 2). Of these, a major QTL, designated as *QLr.ags‐1AL*, was detected in all field and greenhouse adult plant tests with a LOD value of 11.44 to 33.83 (Table 2; Figure 3). *QLr.ags‐1AL* was flanked by *IWB20487* and *IWA4022* markers on the long arm of chromosome 1A and explained up to 34.45% of phenotypic variation in AGS 2038/UGA 111729 RIL population (Table 2). This resistance allele was contributed by resistant parent AGS 2038 (Table 2). A second QTL, *QLr.ags‐2DS*, was detected in all field and greenhouse adult plant tests except Plains 2018 (APR‐PL18) and explained up to 20.62% of the phenotypic variation (Table 2). *QLr.ags‐2DS* was also detected significant for resistance to *P. triticina* race MFGKG at the seedling stage. *QLr.ags‐2DS* was contributed by AGS 2038 and flanked by *IWA3248* and *IWB943* markers (Table 2; Figure 4). A third QTL, designated as *QLr.ags‐2AS*, was detected significant for adult plant LR resistance at Plains 2019 (APR‐PL19) and in the combined data from all environments (APR‐ALL ENV). *QLr.ags‐2AS* was contributed by resistant parent AGS 2038, explained up to 5.52% of phenotypic variation in the RIL population, and flanked by *IWB10896* and *IWB67304* markers (Table 2; Figure 4). Two of three 2B QTL, designated as *QLr.ags‐2BS1* and *QLr.ags‐2BS2*, were detected significant for adult plant LR resistance in Plains 2018 (APR‐PL18) and Greenhouse 2018 (APR‐GH18) environments, respectively, and explained up to 12.82% of phenotypic variation (Table 2). *QLr.ags‐2BS1* and *QLr.ags‐2BS2* were contributed by UGA 111729 and AGS 2038, respectively (Table 2; Figure 4). *QLr.ags‐2BS3* was detected significant for resistance to *P. triticina* race MFGKG at the seedling stage, explained 15.95% of phenotypic variation, and was contributed by the resistant parent AGS 2038.

**TABLE 2:** Summary of quantitative trait loci (QTL) detected significant for seedling and adult plant leaf rust resistance in AGS 2038/UGA 111729 recombinant inbred line population

QTL	Environments^a^	Flanking markers	Peak position (cM)	Peak LOD	*R^2^* (%)^b^	AE^c^
*QLr.ags‐1AL*	APR‐PL18	*IWB20487‐IWA4022*	59.00	33.83	34.45	‐8.86
	APR‐PL19	*IWB20487‐IWA4022*	59.00	15.57	24.33	−9.92
	APR‐GRF19	*IWB20487‐IWA4022*	59.00	18.19	28.94	−7.89
	APR‐GH18	*IWB20487‐IWA4022*	59.00	11.44	13.52	−7.99
	APR‐ALL ENV	*IWB20487‐IWA4022*	59.00	25.19	29.97	−8.57
*QLr.ags‐2DS*	APR‐PL19	*IWA3248‐IWB943*	71.00	9.57	12.25	−7.04
	APR‐GRF19	*IWA3248‐IWB943*	71.00	6.44	8.11	−4.18
	APR‐GH18	*IWA3248‐IWB943*	71.00	19.19	20.62	−9.86
	ASR‐GH20	*IWA3248‐IWB943*	71.00	14.08	17.96	−1.39
	APR‐ALL ENV	*IWA3248‐IWB943*	71.00	16.74	15.93	−6.25
*QLr.ags‐2AS*	APR‐PL19	*IWB10896‐IWB67304*	62.00	4.74	5.52	−4.72
	APR‐ALL ENV	*IWB10896‐IWB67304*	62.00	3.81	3.21	−2.81
*QLr.ags‐2BS1*	APR‐PL18	*IWA2391‐IWB29273*	74.00	4.33	2.73	2.58
*QLr.ags‐2BS2*	APR‐GH18	*IWB7346‐IWA4673*	97.00	12.60	12.82	−7.92
*QLr.ags‐2BS3*	ASR‐GH20	*IWB43459‐IWB63381*	90.00	4.60	15.95	−1.31

a The six environments were field and greenhouse adult plant tests at Plains 2018 (APR‐PL18), Plains 2019 (APR‐PL19), Griffin 2019 (APR‐GRF19), adult plant greenhouse test (APR‐GH18), combined data from all environments adult plant test (APR‐ALL ENV), and the seedling test for the all‐stage resistance (ASR‐GH20).

b Phenotypic variation explained by the QTL.

c AE, additive effect. Negative and positive values indicate that the QTL was contributed by AGS 2038 and UGA 111729, respectively.

### 3.4 Search for candidate genes within the major QTL region

Based on the location of SNP markers, *QLr.ags‐1AL* was physically mapped to the 579,299,114 to 581,830,275 bp region on chromosome 1A (Figure 3). The CS RefSeq annotation v1.0 indicated a total of 76 genes, including 32 high confidence (HC) and 44 low confidence (LC) genes within *QLr.ags‐1AL* locus region (Supplemental Table S5). Of these, 5 genes were disease resistance protein (NBS‐LLR class) family. Additionally, the *QLr.ags‐1AL* region also harbors protein kinase domains i.e., protein kinase family protein, leucine‐rich repeat receptor‐like protein kinase family protein, and protein kinase. All of these NBS‐LRR genes and protein kinase domains could contribute to LR resistance in AGS 2038 and are possible candidate genes for *QLr.ags‐1AL*.

### 3.5 Polymorphism of major QTL linked markers

Two SNP markers flanking the major QTL, *QLr.ags‐1AL*, were converted into user‐friendly allele‐specific primers and tested on a set of 30 wheat lines and parental genotypes to determine their diagnostic value for MAS. However, only one marker, *IWB20487* linked to *QLr.ags‐1AL*, showed clear polymorphism, and is therefore reported here. Of the 30 wheat lines tested, 16 lines were found to carry the AGS 2038 allele and all others produced the UGA 111729 allele (Supplemental Table S1).

## 4 DISCUSSION

QTL mapping using bi‐parental mapping populations is a common and widely used approach to dissect complex traits and to detect genomic regions associated with the quantitative traits in plants (Wang et al., 2015). However, the success and precision of QTL mapping largely depends on the type and size of the population used, marker type, and density of markers used for the construction of linkage map (Chen et al., 2014; Wang et al., 2015). In this study, we used the Infinium iSelect 90K assay (Wang et al., 2014) for genotyping and development of a high‐density linkage map for a RIL population consisting of 225 lines. A high‐density linkage map was developed containing 7,667 polymorphic markers distributed on all 21 chromosomes. The total genetic distance of 2,881.46 cM with marker density of 0.38 cM/marker and 1.90 cM/locus represented an improvement on several linkage maps published earlier (Kumar et al., 2016; Russo et al., 2014; Wen et al., 2018). However, some of the chromosomes i.e., 1A, 4B, 5D, 6D, and 7B, had poor marker coverage (Table 2). One possible reason is that in some of the chromosome regions, the genome of parental lines, AGS 2038 and UGA 111729, could be fixed (no polymorphism) which is evidenced by the fact that both parents possess some level of LR resistance, and therefore, no segregation occurred in the population. Additionally, since Infinium iSelect 90K had poor representation of the D‐genome (Wang et al., 2014), some of the chromosomes (5D and 6D) likely had poor marker coverage.

The utilization of genetic resistance is undoubtedly the most preferred method to control wheat pests in general and rust diseases in particular. Although 79 *Lr* genes and more than 200 QTL have been cataloged for LR resistance, a constant search for novel genes/QTL is necessary to combat new and virulent *P. triticina* races emerging every year worldwide. In this study, we mapped six QTL for seedling and adult plant LR resistance in a AGS 2038/UGA 111729 RIL population using a high‐density linkage map. Of these, a major QTL, *QLr.ags‐1AL*, was consistently detected in all field and greenhouse adult plant tests and physically mapped at 579,299,114 to 581,830,275 bp region on the long arm of chromosome 1A (Figure 3). While AGS 2038 has high level of seedling and adult plant LR resistance, the major QTL, *QLr.ags‐1AL*, was detected only in adult plant tests indicating that it is effective only at adult plant stage.

Chromosome 1A carries two previously known *Lr* genes, *Lr10* (Feuillet, Schachermayr, & Keller, 1997) and *Lr59* (Marais, McCallum, & Marais, 2008) which are located on the short and long arms, respectively. We believe that the major QTL, *QLr.ags‐1AL*, detected in this study for adult plant LR resistance is unique and different from *Lr59*, the only gene mapped on chromosome 1AL, based on the following reasons. Firstly, *Lr59* was derived from *Aegilops peregrine* following an introgression into common wheat. It is thought that the *A. peregrine* introgression replaced the complete long arm of wheat chromosome 1A (Marais et al., 2008; Pirseyedi et al., 2015). Based on the pedigree information, AGS 2038 does not share any parents that appear to contain this alien introgression. Second, alien translocations are accompanied by large linkage blocks which was not observed in our mapping population. Third, based on molecular marker analysis using a diagnostic marker linked to *Lr59*, we confirmed that AGS 2038, donor of *QLr.ags‐1AL*, was negative to *Lr59* (Supplemental Figure S1). Therefore, based on these facts, we believe that *QLr.ags‐1AL* was different than *Lr59* and is temporarily designated as *Lr2K38*.

A minor QTL, *QLr.ags‐2AS*, was detected on the short arm of chromosome 2A and flanked by *IWB10896* and *IWB67304* markers (Table 2; Figure 4). Six known *Lr* genes, *Lr11*, *Lr17*, *Lr37*, *Lr45*, *Lr65*, and *LrAlt* were previously mapped in the vicinity of *QLr.ags‐2AS* (McIntosh et al., 2008; Wang et al., 2010). Of these, *Lr37* and *Lr45* were derived from *A. ventricosa* and *Secale cereale*, respectively (McIntosh, Friebe, Jiang, & Gill, 1995a; Helguera et al., 2003), and therefore, are unlikely to be incorporated in SRWW adopted cultivars. *Lr11* was derived from common wheat cultivar ‘Hussar’ on chromosome 2AS; however, later studies demonstrated that the chromosomal location of *Lr11* was erroneous (Darino et al., 2015). *Lr17* has two resistance alleles i.e., *Lr17*a and *Lr17b* (McIntosh et al., 2008). Based on the wheat consensus genetic map (Maccaferri et al., 2015), *Lr17a* and *Lr65* linked marker (*Xgwm614*) and *LrAlt* linked marker (*Xgwm636*) were mapped 39.69 and 36.51 cM distal to *QLr.ags‐2AS*, respectively, and likely represent different loci for LR resistance. However, further studies such as an allelism test or molecular marker analysis are warranted to determine the relationship between *QLr.ags‐2AS* and other *Lr* genes/QTL previously mapped on chromosome 2AS.

Three QTL, *QLr.ags‐2BS1*, *QLr.ags‐2BS2*, and *QLr.ags‐2BS3*, were detected on chromosome 2BS in the vicinity of seven known *Lr* genes i.e., *Lr13, Lr16, Lr23, Lr48, Lr73, LrZH22*, and *LrA2K* (McIntosh et al., 2008; Park, Mohler, Nazari, & Singh, 2014; Sapkota et al., 2019a, 2019b; Wang et al., 2016). Of these, *Lr48* linked marker (*Xgwm429b*) was located closest to *QLr.ags‐2BS1* (6.38 cM proximal), based on the wheat consensus map (Maccaferri et al., 2015), and likely represent the same locus since both confer adult plant LR resistance. However, further studies are warranted to determine the relationship between *QLr.ags‐2BS1* and other previously mapped *Lr* genes. *QLr.ags‐2BS2* (adult plant resistance QTL) and *QLr.ags‐2BS3* (seedling resistance QTL) were mapped very close on 2BS chromosome (7 cM apart) and likely represent a single locus for LR resistance. These two loci were detected on the same location where we detected *LrA2K* in our previous study (Sapkota et al., 2019b). Furthermore, molecular marker analysis demonstrated that AGS 2038 was positive for the *LrA2K* linked marker *Xwmc770* (Sapkota et al., 2019b). Therefore, *QLr.ags‐2BS2, QLr.ags‐2BS3*, and *LrA2K* most likely represent the same locus for LR resistance. Of the three QTL detected on 2BS chromosome, two QTL (*QLr.ags‐2BS2* and *QLr.ags‐2BS3*) were contributed by AGS 2038 while *QLr.ags‐2BS1* was contributed by the UGA 111729. The reasons that both parents contributed QTL for LR resistance probably accounted for the fact that both parents had some level of LR resistance and therefore, transgressive segregation was observed among the RILs.

QTL *QLr.ags‐2DS*, detected significant for both seedling and adult plant LR resistance in AGS 2038, was mapped in the vicinity of three known *Lr* genes, *Lr2*, *Lr22*, and *Lr39* (McIntosh et al., 2008; Raupp, Sukhwinder, Brown‐Guedira, & Gill, 2001). *Lr2* has three different alleles, *Lr2a, Lr2b*, and *Lr2c*, and is linked to the centromere region of the 2D chromosome (McIntosh, Wellings, & Park, 1995b). *Lr22* has two alleles, *Lr22a* and *Lr22b*. *Lr22a* was transferred to wheat from *Ae. tauschii* (Dyck, 1979; Raupp et al., 2001). *Lr39* confers both seedling and adult plant leaf rust resistance and was transferred to wheat from *Ae. tauschii* (Raupp et al., 2001) and is unlikely to be present in adapted SRWW wheat. However, further work is needed to verify the relationship between *QLr.ags‐2DS* and other known *Lr* genes/QTL previously mapped on chromosome 2DS.

Two SNP markers that were tightly linked to *Lr2K38* gene were converted into user‐friendly allele‐specific primers and used to genotype a set of 30 wheat materials to test their diagnostic value for MAS. All wheat lines that were rated as resistant and susceptible to LR at adult plant stage were found to carry the resistant and susceptible allele, respectively, with a few exception (Supplemental Table S1). Two SRWW cultivars, USG 3024 and GW 2032, were phenotypically LR resistant but were negative to resistance allele (Supplemental Table S1) indicating that they likely carry different LR resistant genes. SRWW breeding line GA131214‐4‐3‐2 was found to carry resistant allele but was phenotypically susceptible (Supplemental Table S1) indicating that this marker confers varying level of resistance based on what other gene combination likely present. Similarly, three breeding lines, 410‐18E11, 411‐18E12, and 423‐18E25, that had AGS 2038 in their pedigree produced resistance allele (Supplemental Table S1). Overall, this result showed that the marker *IWB20487* is highly effective for MAS and pyramiding *Lr2K38* gene.

Different types of disease resistance genes (R‐genes) have been reported in plants; however, the majority of R‐genes cloned to date encode nucleotide‐binding site leucine‐rich repeat (NBS‐LRR) proteins (Adhikari & Missaoui, 2019; McHale, Tan, Koehl, & Michelmore, 2006). Among the 79 genes for LR resistance that have been cataloged so far, only 6 *Lr* genes, *Lr1, Lr10, Lr21, Lr22a, Lr34*, and *Lr67* have been cloned (Cloutier et al., 2007; Feuillet et al., 2003; Huang et al., 2003; Krattinger et al., 2009; Moore et al., 2015; Sapkota et al., 2019b; Thind et al., 2017). Of these*, Lr1, Lr10*, and *Lr21* encode for NBS‐LRR proteins (Cloutier et al., 2007; Feuillet et al., 2003; Huang et al., 2003). Most of the R‐genes reported in wheat‐rust pathosystems confer race‐specific resistance in the gene‐for‐gene model and have been cloned as NBS‐LRR proteins (Krattinger & Keller, 2016). In this study, we detected 5 NBS‐LRR and several kinase‐related genes in the *Lr2K38* region and most likely these genes play an important role in conferring LR resistance in AGS 2038. Similar to our study, Wang et al. (2019) and Zhou et al. (2019) also reported NBS‐LRR and kinase‐related genes within the QTL region detected for dwarf bunt and stripe rust resistance, respectively, in wheat. The candidate genes detected within the *Lr2K38* region will be valuable in fine mapping and cloning of *Lr2K38*. The sequence comparison between the parents, AGS 2038 and UGA 111729, for these candidate genes could be helpful in identifying the actual gene(s) responsible for LR resistance.

In summary, we detected 6 QTL for seedling and/or adult plant LR resistance in wheat utilizing a RIL population consisting of 225 lines and 90K SNP genotyping data. A novel adult plant LR resistance QTL, *QLr.ags‐1AL* (*Lr2K38*), was detected on the long arm of chromosome 1A with major effect (Table 2, Figure 3). Based on the candidate gene search in the *Lr2K38* region (IWGSC RefSeq Annotations V1.0), 5 NBS‐LRR related genes were annotated and are possible candidate genes for the *Lr2K38* locus. *Lr2K38* linked marker *IWB20487* was highly polymorphic among the SRWW cultivars and can be used for MAS of *Lr2K38* in wheat breeding programs. Adult plant LR resistance in SRWW cultivar AGS 2038 is highly effective against current *P. triticina* races in the Southeastern US, and therefore, pyramiding *Lr2K38* with other *Lr* genes, such as *Lr34* and *Lr68*, could provide long lasting LR resistance.

## AUTHOR CONTRIBUTIONS

M. Mergoum and J. Johnson designed the study; S. Sapkota, D. Bland, B. Lopez, S. Sutton, B. Ghimire, J. Buck, and S. Harrison phenotyped the population; S. Sapkota, A. Kumar, and J. Fiedler genotyped the population and construct the linkage map; S. Sapkota and Z. Chen developed primers and conducted assays, S. Sapkota analyzed the data and wrote the manuscript; M. Mergoum supervised the entire study; M. Mergoum and J. Buck edited the manuscript.

## CONFLICT OF INTEREST

All authors read the manuscript and declare that there is no conflicts of interest.

## Supporting information

Supplemental Figure S1: Ethidium bromide stained agarose gel showing the amplification of SCAR marker S15‐T3 on parental lines AGS 2038 (A) and UGA 111729 (U). The 100 bp DNA ladder (L) was added to determine the size of the amplified products. The S15‐T3 marker amplified a 622 bp band diagnostic for *Lr59* gene. Both parents, AGS 2038 and UGA 111729, did not produce a 622 bp band.

Supplemental Table S6: Phenotypic data (seedling and adult plant test) of the AGS 2038/UGA 111729 derived RIL population used for QTL analysis
