# Supplementary material for: A novel adult plant leaf rust resistance gene Lr2K38 mapped on wheat chromosome 1AL
Source: Plant Genome. 2020 Nov 10;13(3):e20061. doi: 10.1002/tpg2.20061 (PMC12807417; doi:10.1002/tpg2.20061)
Supplement: Supplementary file 1 [file TPG2-13-e20061-s001.docx]

**Table S1** List of soft-red winter wheat (SRWW) lines used to test the polymorphism of resistance linked marker.

| **S. N** | **Line Name** | **Pedigree** | **Phenotype**^‡^ | ***IWB20487***^§^ |
| --- | --- | --- | --- | --- |
| 1 | GA131216-1-4 | JAMESTOWN/031257-10LE34//051477-9-1-6 | S | CC |
| 2 | GA13011ID-2-6 | JAMESTOWN/031257-10E41 | S | CC |
| 3 | GA13085-9-6 | 06478-23-1-3/AGS2033 | R | TT |
| 4 | GA13264-1-6 | JAMESTOWN/AGS2033 | S | CC |
| 5 | GA131187-3-9-2 | 061098-9-6-1/SH5550 | R | TT |
| 6 | GA15020ID-7-7 | JT152/VA11W-230//04417-12E33 | S | CC |
| 7 | GA14317ID-11-5 | JT152/PIO26R94 | R | TT |
| 8 | GA141205ID-20-2 | HILLIARD/PIO26R94 | S | CC |
| 9 | GA131121-4-3-1 | 05153-16-2-4/031086-10E26//AGS2033 | R | TT |
| 10 | GA131214-4-3-2 | LA06146E-P05/JAMESTOWN/051477-9-1-6 | S | TT |
| 11 | GA131397-6-4-7 | 04494-11E49/031257-10E41//041293-11E37 | R | TT |
| 12 | GA131514-5-2-6 | JT/031238-7E34//051477-9-1-6 | S | CC |
| 13 | GA131700-2-2-12 | PIO26R20/04570-10E46/AGS*2/021773 | R | TT |
| 14 | GA12212-10-4-4 | 041293-11LE37/031134-10E29 | R | TT |
| 15 | GA14065I-14-3-7 | 06385-13-2-3/031257-10LE34 | R | TT |
| 16 | GA14119I-9-2-6 | VA10W-119/041293-11LE37//041293-11E54 | S | CC |
| 17 | GA14186I-15-3-9 | 041293-11LE37/06399-G2-4-1//04434-11E44 | R | TT |
| 18 | GA14187I-4-3-4 | VA10W-119/04570-10E46//041293-11E54 | S | CC |
| 19 | GA14230I-6-4-8 | 04417-12E33/041052-11E51//VA10W-123 | R | TT |
| 20 | GA14329I-17-2-3 | TV8861/04434-11E44//04434-11E44 | R | TT |
| 21 | GA14119I11-2-2 | VA10W-119/11LE37//SH5550 | S | CC |
| 22 | GA131214-8-2-1 | AGS3000//JT//051477-9-1-6 | S | CC |
| 23 | 410-18E11 | 041333-13-2 /AGS2038 | R | TT |
| 24 | 411-18E12 | 021338-9E11/AGS2038 | R | TT |
| 25 | 423-18E25 | AGS2038/VA04W-90 | R | TT |
| 26 | AGS 2035 | 89482-E7/XW663 | R | TT |
| 27 | USG 3024 | SS 8641/961591-17-1-5 | R | CC |
| 28 | GW 2032 | SS 8641 / Oglethorpe // 991371-6E13 | R | CC |
| 29 | USG3555 | VA94-52-60/Pio2643//USG3209 | S | CC |
| 30 | AGS 3000 | Jamestown/AGS 2060 | S | CC |

^‡^ The phenotypic data of the SRWW lines are obtained from the University of Georgia small grain breeding program. R = resistant phenotype; S = susceptible phenotype.

^§^ “TT” and “CC” indicates AGS 2038 and UGA 111729 allele, respectively.

**Table S2** Primer and probe sequences of two SNP markers linked to *QLr.ags-1AL* on chromosome 1AL in wheat

| **SNP marker** | **Primer/Probe Sequences** | |
| --- | --- | --- |
| *IWB20487* | Forward Primer | CCGTCGATTTGGAACAATCTC |
|  | Reverse Primer | TGGGAAAATTAACTACCAGGAGTTTG |
|  | Probe1 | TGTTATTCTTCATCATCGC |
|  | Probe2 | TGTTATTCTTCATCATTGC |
| *IWA4022* | Forward Primer | TGAATAAGACTTTATGAGTCAGGCAAA |
|  | Reverse Primer | GCCCATGTCGATATATCCTACTACTG |
|  | Probe1 | ATTACTTTCCGAAGCGAGA |
|  | Probe2 | TACTTTCCGAAGCAAG |

**Table S3** Analysis of variance (ANOVA) for leaf rust severity data in AGS 2038/UGA 111729 derived recombinant inbred line population.

| Source of variation | Degrees of freedom (DF) | *F*-value | P > F |
| --- | --- | --- | --- |
| Genotype (G) | 224 | 25.28 | <.0001 |
| Environment (E) | 3 | 216.25 | <.0001 |
| G × E | 668 | 2.42 | <.0001 |

**Table S4** Summary of the seedling test data of AGS 2038/UGA 111729 derived recombinant inbred line (RIL) population.

| *P. triticina* | Parental lines | |  | RIL population | | | | | |
| --- | --- | --- | --- | --- | --- | --- | --- | --- | --- |
|  | AGS 2038 | UGA 111729 |  | Mean | Min. | Max. | Std. Dev. | *H^2^* |  |
| MFGKG^a^ | 1.0 | 8.0 |  | 5.9 | 0.0 | 9.0 | 3.2 | 0.9 |  |

*H^2^* = broad-sense heritability

^a^AGS 2038/UGA 111729 derived RIL population was phenotyped at the seedling stage with *P. triticina* race MFGKG. The 0-4 infection type (IT) scale was converted to a linearized scale of 0-9 and used for QTL analysis.

**Table S5** Gene annotations within the candidate region of the major QTL identified in AGS 2038/UGA 111729 population

| **Gene ID** | **Chrom.** | **Position (bp)** | **Human-Readable-Description** |
| --- | --- | --- | --- |
| TraesCS1A01G424300.1 | 1A | 579300387 | Protein ABIL1 |
| TraesCS1A01G424400.1 | 1A | 579533509 | S-type anion channel |
| TraesCS1A01G424500.1 | 1A | 579573900 | S-type anion channel |
| TraesCS1A01G424600.1 | 1A | 579647984 | TTF-type zinc finger protein with HAT dimerization domain-containing protein |
| TraesCS1A01G424700.1 | 1A | 579677386 | F-box family protein |
| TraesCS1A01G424800.1 | 1A | 579836462 | Cellulose synthase |
| TraesCS1A01G424900.1 | 1A | 579861533 | Protein kinase family protein |
| TraesCS1A01G425000.1 | 1A | 579864314 | Mitochondrial carrier-like protein |
| TraesCS1A01G425100.1 | 1A | 580183361 | Indole-3-acetic acid-amido synthetase GH3.3 |
| TraesCS1A01G425200.1 | 1A | 580420857 | Extra-large G-like protein |
| TraesCS1A01G425300.1 | 1A | 580433890 | Amino acid transporter family protein |
| TraesCS1A01G425400.1 | 1A | 580444570 | Amino acid transporter family protein |
| TraesCS1A01G425500.1 | 1A | 580448575 | Amino acid transporter family protein |
| TraesCS1A01G425600.1 | 1A | 580453354 | transmembrane protein, putative (DUF594) |
| TraesCS1A01G425700.1 | 1A | 580492051 | Transducin/WD40 repeat protein |
| TraesCS1A01G425800.1 | 1A | 580571333 | Disease resistance protein (NBS-LRR class) family |
| TraesCS1A01G425900.1 | 1A | 580659531 | Disease resistance protein (NBS-LRR class) family |
| TraesCS1A01G426000.1 | 1A | 580739950 | Disease resistance protein (NBS-LRR class) family |
| TraesCS1A01G426100.1 | 1A | 580925443 | Lipid transfer protein |
| TraesCS1A01G426200.1 | 1A | 580959561 | B-block binding subunit of TFIIIC |
| TraesCS1A01G426300.1 | 1A | 580988276 | Polyol transporter |
| TraesCS1A01G426400.1 | 1A | 581206186 | Agenet and bromo-adjacent homology (BAH) domain-containing protein |
| TraesCS1A01G426500.1 | 1A | 581255339 | Agenet and bromo-adjacent homology (BAH) domain-containing protein |
| TraesCS1A01G426600.1 | 1A | 581256211 | Ribonucleoside-diphosphate reductase large subunit |
| TraesCS1A01G426700.1 | 1A | 581438178 | RNA polymerase sigma factor |
| TraesCS1A01G426800.1 | 1A | 581506492 | Polypyrimidine tract-binding-like protein, putative |
| TraesCS1A01G426900.1 | 1A | 581540710 | Pentatricopeptide repeat-containing protein, putative |
| TraesCS1A01G427000.1 | 1A | 581570414 | Protein phosphatase 2C family protein |
| TraesCS1A01G427100.1 | 1A | 581580561 | CTP synthase |
| TraesCS1A01G427200.1 | 1A | 581589503 | Protein N-methyltransferase |
| TraesCS1A01G427300.1 | 1A | 581754085 | Leucine-rich repeat receptor-like protein kinase family protein |
| TraesCS1A01G427400.1 | 1A | 581829492 | TPR repeat-containing thioredoxin TTL1 |
| TraesCS1A01G601100LC.1 | 1A | 579584639 | Gag polyprotein |
| TraesCS1A01G601200LC.1 | 1A | 579647403 | Zinc finger MYM-type-like protein |
| TraesCS1A01G601300LC.1 | 1A | 579712406 | Tryptophan synthase beta chain |
| TraesCS1A01G601400LC.1 | 1A | 579762850 | 60S ribosomal protein L30 |
| TraesCS1A01G601500LC.1 | 1A | 579785979 | LINE-1 reverse transcriptase like |
| TraesCS1A01G601600LC.1 | 1A | 579826503 | Transcription elongation factor Spt5 |
| TraesCS1A01G601700LC.1 | 1A | 579830044 | Formyl-CoA:oxalate CoA-transferase |
| TraesCS1A01G601800LC.1 | 1A | 579843454 | RNA-binding (RRM/RBD/RNP motifs) family protein |
| TraesCS1A01G601900LC.1 | 1A | 579843550 | 4F5 protein family protein |
| TraesCS1A01G602000LC.1 | 1A | 579937793 | Calcium-dependent lipid-binding (CaLB domain) family protein |
| TraesCS1A01G602100LC.1 | 1A | 580167995 | Protein FAR1-RELATED SEQUENCE 5 |
| TraesCS1A01G602200LC.1 | 1A | 580174146 | Basic-leucine zipper (bZIP) transcription factor family protein |
| TraesCS1A01G602300LC.1 | 1A | 580417134 | Glutathione S-transferase T3 |
| TraesCS1A01G602400LC.1 | 1A | 580576844 | Retrotransposon protein, putative, LINE subclass |
| TraesCS1A01G602500LC.1 | 1A | 580579258 | Endonuclease/exonuclease/phosphatase family protein |
| TraesCS1A01G602600LC.1 | 1A | 580579519 | Cyclin/Brf1-like TBP-binding protein |
| TraesCS1A01G602700LC.1 | 1A | 580580191 | Heat shock 70 kDa protein |
| TraesCS1A01G602800LC.1 | 1A | 580582853 | Disease resistance protein (NBS-LRR class) family |
| TraesCS1A01G602900LC.1 | 1A | 580627181 | Histidine--tRNA ligase |
| TraesCS1A01G603000LC.1 | 1A | 580628996 | Disease resistance protein (NBS-LRR class) family |
| TraesCS1A01G603100LC.1 | 1A | 580637490 | Sushi, von Willebrand factor type A, EGF and pentraxin domain-containing protein 1 |
| TraesCS1A01G603200LC.1 | 1A | 580656814 | LINE-1 reverse transcriptase |
| TraesCS1A01G603300LC.1 | 1A | 580657514 | RNA-directed DNA polymerase (reverse transcriptase)-related family protein |
| TraesCS1A01G603400LC.1 | 1A | 580658164 | JmjC domain protein JMJ24 |
| TraesCS1A01G603500LC.1 | 1A | 580738529 | Vacuolar sorting ATPase |
| TraesCS1A01G603600LC.1 | 1A | 580746488 | Retrotransposon protein, putative, unclassified |
| TraesCS1A01G603700LC.1 | 1A | 580893424 | splicing factor PWI domain-containing protein / RNA recognition  motif (RRM)-containing protein |
| TraesCS1A01G603800LC.1 | 1A | 580940247 | microtubule-associated proteins 65-1 |
| TraesCS1A01G603900LC.1 | 1A | 580941502 | B-block binding subunit of TFIIIC |
| TraesCS1A01G604000LC.1 | 1A | 580947068 | LINE-1 reverse transcriptase |
| TraesCS1A01G604100LC.1 | 1A | 580988871 | Ribonuclease Z |
| TraesCS1A01G604200LC.1 | 1A | 581264937 | O-fucosyltransferase family protein |
| TraesCS1A01G604300LC.1 | 1A | 581449872 | No-apical-meristem-associated carboxy-terminal domain protein |
| TraesCS1A01G604400LC.1 | 1A | 581465447 | RNA-directed DNA polymerase (Reverse transcriptase) |
| TraesCS1A01G604500LC.1 | 1A | 581511230 | Acetyl-CoA carboxylase 1 |
| TraesCS1A01G604600LC.1 | 1A | 581512723 | LINE-1 reverse transcriptase-like protein |
| TraesCS1A01G604700LC.1 | 1A | 581569578 | Tetratricopeptide repeat (TPR)-like superfamily protein |
| TraesCS1A01G604800LC.1 | 1A | 581593927 | Protein kinase |
| TraesCS1A01G604900LC.1 | 1A | 581637956 | Ribosomal protein L11 methyltransferase |
| TraesCS1A01G605000LC.1 | 1A | 581638454 | Zinc transporter 5 |
| TraesCS1A01G605100LC.1 | 1A | 581640879 | Methionine S-methyltransferase |
| TraesCS1A01G605200LC.1 | 1A | 581667710 | Far1-like |
| TraesCS1A01G605300LC.1 | 1A | 581678836 | Transposon protein, putative, Mutator sub-class |
| TraesCS1A01G605400LC.1 | 1A | 581748487 | Transposon protein, putative, CACTA, En/Spm sub-class |

**Fig. S1** Ethidium bromide stained agarose gel showing the amplification of SCAR marker S15-T3 on parental lines AGS 2038 (A) and UGA 111729 (U). The 100 bp DNA ladder (L) was added to determine the size of the amplified products. The S15-T3 marker amplified a 622 bp band diagnostic for *Lr59* gene. Both parents, AGS 2038 and UGA 111729, did not produce a 622 bp band.
